# The prevalence of musculoskeletal pain among above 50-year-old population referred to the Kermanshah-Iran health bus in 2016

**DOI:** 10.1186/s13104-020-4940-6

**Published:** 2020-02-12

**Authors:** MohammadBagher Shamsi, Ameneh Safari, Soheila Samadzadeh, Nasrin Yoosefpour

**Affiliations:** 1 School of Allied Medical Sciences, Kermanshah University of Medical Sciences, DowlatAbad Street, Kermanshah, Iran; 2 Department of Radiology, School of Allied Medical Sciences, Kermanshah University of Medical Sciences, Kermanshah, Iran; 3 Department of Rehabilitation and Sports Medicine, School of Medicine, Kermanshah University of Medical Sciences, Kermanshah, Iran

**Keywords:** Pain, Musculoskeletal disorders, Elderly

## Abstract

**Objective:**

Musculoskeletal disorders are one of the most important causes of disability. The aim of this study was to evaluate the prevalence of musculoskeletal disorders among the above 50-year-old population who referred to the health bus in Kermanshah. A questionnaire was used to collect data regarding musculoskeletal disorders of 589 people who voluntarily referred to the health bus. Means (SD) and range were used in descriptive statistics.

**Results:**

The most prevalence of musculoskeletal complaints was related to the knee (338, 57.4%). After that, the low back pain had the most frequency (319, 54.3%). The lowest prevalence of musculoskeletal complaints was related to the thighs with a frequency of 95 (16.1%). The highest rate of musculoskeletal complaints over the past week in both genders with a frequency of 251 (59.3%) among women and 68 (41%) among men was related to the low back region The highest rate of musculoskeletal complaints over the past year was also related to the low back region, with a frequency of 220 (41%) among women and 61 (36.7%) among men. Concerning the physical health of the elderly, special attention is required for the knee joint and the low back region.

## Introduction

Musculoskeletal disorders (MSDs) are one of the most common and costly problems in the world [1]. The economic costs of these disorders not only affect the individual but also society [2]. As estimated, the costs of musculoskeletal disorders are about 1% of the industrialized country’s gross national products [3].

Musculoskeletal disorders are a multi-factorial phenomenon whose risk factors can be grouped into 4 categories of genetic, anatomical, psychosocial and biomechanical factors [4, 5]. According to a report from the National Center for Finland Statistics in 1998, 46% of all diseases are related to musculoskeletal disorders, which occur in various areas of the body such as the neck, shoulder, elbow, hand, low back and hip joints [6]. Musculoskeletal disorders account for 0.7 of all diseases in the community, 0.14 of those referred people to physicians and 0.19 of hospitalization cases [7]. According to the fact that many cases of musculoskeletal injuries are not reported, so the existing figures are like an iceberg peak, which only shows the appearance of the problem and does not specify its extent [8]. Various studies have shown that older men and women are more likely to be injured and impaired than young people, and complaints of musculoskeletal disorders are more in older adults [9–11].

In addition to the aging factor, studies have shown that the occupation and gender of individuals can also be effective in musculoskeletal pain [12–15]. For example, Kalian et al. in a study found that older men and women are more likely to be injured and impaired than young people [9]. Considering the aging as one of the risk factors for the prevalence of musculoskeletal problems [11] and, on the other hand, paying attention to the growth of the population over the age of 50 and movement of the countries to the aging, particular attention to this demographic group and their health needs are considered essential. Accordingly, as there has not been a study on this population group in Iran, this study was conducted. The aim was assessing the health status of the musculoskeletal system in a population over 50 years old referring to the Kermanshah (a city in Iran) health bus. It was in order to provide an overview of the current status of these people in Kermanshah for the prevalence of musculoskeletal disorders and to be useful in interventional plans for reducing musculoskeletal disorders.

## Main text

### Methods

This was a descriptive cross-sectional study. Using Davatchi et al.’s study [16], with a confidence interval of 95 and 5% level of significance, the sample size was calculated to be 588. The research community of the study consisted of 589 people who referred to the health bus in Kermanshah in 2016, who were selected by the available sampling method. A questionnaire was used to collect data during an interview. So many eligible people were taken until attaining the required number of participants. The questionnaire of this study consisted of three main parts: in the first part, demographic data such as age, gender, marital status, educational level, and body mass index (BMI), and etc. were recorded. BMI was divided into four groups: underweight (BMI less than 18.5), normal (BMI between 18.4 and 24.9), overweight (BMI between 25 and 30), and obese (BMI > 30). BMI was calculated by dividing the weight in kilograms by the height in meters squared. In the second part, the Nordic Musculoskeletal Questionnaire was used to determine the location of musculoskeletal disorders. Using this questionnaire, the respondent should determine which of the nine parts of his body (neck, shoulder, elbow, hand, upper back, low back, thigh/hip, knee, and leg/ankle) has suffered during the last 12 months. Subsequently, the respondent should have been questioned whether this problem caused her/him to leave the job or her/his inability in working or not. Furthermore, the reduction of work activity and leisure activity due to musculoskeletal pain were questioned. Furthermore, the information of any pain or discomfort in the past 7 days for each of these areas was inquired. The Nordic questionnaire was developed and implemented by Koran and colleagues in 1987 at the Institute of Occupational Health in Scandinavia with the aim of determining the prevalence of musculoskeletal disorders caused by work. The validity and reliability of the Persian version of this questionnaire have been confirmed in previous studies [8]. The inclusion criteria included age over 50 years old, lack of congenital and primary musculoskeletal disorders, lack of history of bone surgery, and the exclusion criteria was a reluctance to cooperate during the study. To complete the questionnaires, Kermanshah city was divided into eight municipal districts and based on the weekly scheduled plan, researchers had to visit a neighborhood with a health bus and had to stay in that neighborhood for 1 week. The people who voluntarily referred to the health bus to assess their health status, besides that, their musculoskeletal system was evaluated and a Nordic questionnaire was completed for them. Each person was asked whether she/he had any problem or pain in different parts of the body over 12 months ago and whether these problems have caused leaving the job or inability in doing the job or not. It was also asked about pain or disorder in each of these parts in the past 7 days. Means (SD) and range were used for descriptive statistics. Data were analyzed using SPSS 18.

## Results

In this study, 589 individuals who referred to the health bus in Kermanshah were evaluated, who 71.8% (423 individuals) were women. Regarding the educational level, the highest frequency was related to having a high school degree (74%) and the lowest (8%) was related to having an academic degree.

The results showed that the highest occurrence of musculoskeletal disorders with the frequency of 338 individuals (57.4%) was related to the knee. Then, the low back pain had the highest frequency (319, 54.2%). The lowest number of musculoskeletal disorders (95, 16.1%) was related to the thigh.

According to the findings in Table 1, which shows the prevalence of musculoskeletal pain by part of the body and gender of the studied people, the highest occurrence of musculoskeletal disorders among women, over the last year, was related to their knees (65.9%) and low back (59.3%), and the highest occurrence of musculoskeletal disorders among men was related to the low back (41%) and then knees (36.1%). The prevalence of musculoskeletal pain among men and women in the last week follows a similar pattern.

**Table 1:** Frequency distribution of the prevalence of musculoskeletal disorders divided by part of body in the last year and last week among people above 50-Year-Old

Having pain in recent 12 month (number (%))				Having pain in recent week (number (%))				Body part
Male		Female		Male		Female		
No	Yes	No	Yes	No	Yes	No	Yes	
138 (83.1%)	28 (16.9%)	262 (61.9%)	161 (38.1%)	131 (78.9%)	35 (21.1%)	233 (55.1%)	190 (44.9%)	Neck
133 (80.1%)	33 (19.9%)	261 (61.7%)	162 (38.3)	122 (73.5%)	44 (26.5%)	222 (52.5%)	201 (47.5%)	Shoulder
149 (89.8%)	17 (10.2%)	338 (79.9%)	85 (20%)	146 (88.0%)	20 (12.0%)	319 75.4%)	104 (24.6%)	Elbow
148 (89.2%)	18 (10.8%)	279 (65.9%)	144 (34.1%)	140 (84.3%)	26 (15.7%)	259 (61.3%)	164 (38.7%)	Hand/Wrist
149 (89.8%)	17 (10.2%)	285 (67.1%)	139 (32.9%)	142 (85.5%)	24 (14.5%)	249 (58.9%)	174 (41.1%)	Upper Back
105 (63.3%)	61 (36.7%)	203 (48%)	220 (41%)	98 (59%)	68 (41%)	172 (40.7%)	251 (59.3%)	Low Back
148 (89.2%)	18 (10.8%)	364 (86.1%)	59 (13.9%)	145 (87.4%)	21 (12.6%)	347 (82%)	76 (18%)	Thigh/Hip
129 (77.7%)	37 (22.3%)	206 (48.7%)	217 (51.3%)	106 (63.9%)	60 (36.1%)	144 (34.1%)	279 (65.9%)	Knee
147 (88.6%)	19 (11.4%)	318 (75.2%)	105 (24.8%)	141 (84.9%)	25 (15.1%)	298 (70.4%)	125 (29.6%)	Ankle/Leg

The findings of the research (Additional file 1: Figure S1) indicated that most of the workplace accidents resulted in musculoskeletal disorders among the participants of this study, was in the low back (15 individuals, 2.54%), and the lowest workplace accidents were in the shoulder area (7 individuals, 1.18%). According to these findings, based on the age range of the participant, the highest workplace accidents in the low back region were between 50 and 60, in the neck between 20–30, 30–40 and 40–50, in the shoulder 40–50, and in hand 50–60.

The results of the study showed that in total, 13 participants (2.3%) had changed their job due to musculoskeletal disorders. Of these, 8 (1.4%) due to neck problems, 3 (0.5%) due to shoulder problems, 1 (0.2%) due to low back problems and 1 (0.2%) due to hand problems had changed their jobs.

Furthermore, the findings showed that more than 35% of the participants have experienced severe or very serious pain in the low back, 23% in the neck, 22% in the shoulder, and 17% in hand.

The results showed that the highest reduction of work activity (2.5%) and leisure activity (30.7%) was related to the pain and disorder in the low back, and the lowest reduction of work activity and leisure activity was due to the musculoskeletal problems in the hands (respectively, 0.3% and 17.3%) (Table 2).

**Table 2:** Reduced work activity and leisure activity due to musculoskeletal problems in different parts of the body over the past 12 months

Reduces daily recreation	Reduce occupational activity	Body parts
131 (22.2%)	8 (1.4%)	Neck
140 (23.8%)	6 (1.0%)	Shoulder
181 (30.7%)	15 (2.5%)	Low Back
102 (17.3%)	2 (0.3%)	Hand/Wrist

The results of the disorder prevalence survey in terms of people’s occupation (Table 3) showed that the highest occurrence of musculoskeletal disorders among the housewives was in the low back (237, 40.3%) and knees (262, 44.6%).

**Table 3:** Frequency distribution of the prevalence of musculoskeletal disorders in different parts of the body in the last year based on the occupation of people over 50

Having pain in recent 12 month (number (%))														Occupation/body part
Employee		Drive		Housekeeper		Teacher		Military		Self-employed		Worker		
No	Yes	No	Yes	No	Yes	No	Yes	No	Yes	No	Yes	No	Yes	
16 (2.7%)	8 (1.4%)	14 (2.4%)	3 (0.5)	213 (36.2%)	181 (30.7%)	27 (4.6%)	6 (1%)	29 (4.9%)	4 (0.7)	42 (7.1%)	10 (1.7)	23 (3.9%)	13 (2.2)	Neck
17 (2.9)	7 (1.2)	14 (2.4)	3 (0.5)	204 (34.7%)	190 (32.3%)	23 (3.9)	10 (1.6)	27 (4.6%)	6 (1%)	39 (6.6%)	13 (2.2%)	20 (3.4%)	16 (2.7%)	Shoulder
20 (3.4%)	4 (0.7)	15 (2.5)	2 (0.3)	297 (50.4%)	97 (16.5%)	28 (4.8)	5 (0.8)	31 (5.3)	2 (0.3)	47 (8%)	5 (0.8)	27 (4.6)	9 (1.6)	Elbow
18 (3%)	6 (1)	14 (2.4)	3 (0.5)	241 (41%)	153 (26%)	25 (4.2)	8 (1.4%)	31 (5.3)	2 (0.3)	45 (7.6)	7 (1.2)	25 (4.2)	11 (1.9)	Hand/Wrist
19 (3.2)	5 (0.8)	16 (2.7%)	1 (0.2)	225 (38.2%)	169 (28.7%)	28 (4.8)	5 (0.8)	29 (4.9)	4 (0.7)	46 (7.8)	6 (1%)	28 (4.8)	8 (1.4%)	Upper Back
12 (2)	12 (2)	12 (2)	5 (0.8)	157 (26.7%)	237 (40.3%)	24 (4.1)	9 (1.6)	17 (2.9)	16 (2.7%)	29 (4.9)	23 (3.9)	19 (3.2)	17 (2.9)	Low Back
21 (3.6)	3 (0.5)	16 (2.7%)	1 (0.2)	320 (54.3%)	74 (12.6%)	31 (5.3)	2 (0.3)	28 (4.8)	5 (0.8)	47 (8%)	5 (0.8)	29 (4.9)	7 (1.2)	Thigh/Hip
12 (2)	12 (2)	15 (2.5)	2 (0.3)	132 (22.4%)	262 (44.6%)	24 (4.1)	9 (1.6)	21 (3.6%)	12 (2)	34 (5.8)	18 (3%)	12 (2)	24 (4.1)	Knee
19 (3.2)	5 (0.8)	14 (2.4%)	3 (0.5)	276 (46.9%)	118 (20.2%)	29 (4.9%)	4 (0.7)	27 (4.6%)	6 (1%)	47 (7.8%)	5 (0.8)	27 (4.6%)	9 (1.6)	Ankle

The results of the study on the prevalence of musculoskeletal disorders in different parts of the body in the last year and last week in the participants’ BMI groups (Additional file 2: Table S1) showed that in participants with BMI > 30, the highest incidence of musculoskeletal disorders over the last year was in knee (129, 22.2%) and low back (115, 19.8%). Furthermore, in this group, the highest incidence of musculoskeletal disorders over 1 week was in the knee (103, 17.8%) and low back (100, 17.2%).

The prevalence of musculoskeletal disorders in different parts of the body in the last year and level of education, and reduction of work activity is demonstrated in Additional file 3: Table S2.

In illiterate participants the highest rates of musculoskeletal disorders over the last year were related to knee (n = 141, 76.6%), hip (n = 91, 49.9%), shoulder (n = 90, 48.9%), and neck (n = 86, 46.8%), respectively. In addition, in non-diploma participants the highest rates of musculoskeletal disorders over the last year were related to knee (n = 63, 56.8%), hip (n = 58, 52.3%), shoulder (n = 51, 45.9%), and neck (n = 39, 35.1%), respectively. In diploma participants the highest rates of musculoskeletal disorders over the last year were related to knee (n = 21, 44.7%), hip (n = 21, 44.7%), neck (n = 16, 34%), shoulder (n = 15, 31.9%), and foot (n = 14, 29.8%), respectively. In participants with upper-diploma the highest rates of musculoskeletal disorders over the last year were related to hip (n = 12, 38.7%), knee (n = 11, 35.5%), shoulder (n = 7, 22.6%), wrist (n = 7, 22.6%), and foot (n = 6, 19.4%), respectively.

There was a significant association between the levels of education and the 12-month prevalence of musculoskeletal disorders in the knee, back, wrist, elbow, shoulder, and neck (all p value < 0.05).

In addition, the results show no association between the 12-month prevalence of musculoskeletal disorders and the reduction of work activity (all p value > 0.05).

## Discussion

With regard to the importance of prevention and treatment of musculoskeletal disorders and the effect of these problems on the effectiveness of the society and its workforce, this study was done with the aim of evaluating the prevalence of musculoskeletal system disorders in patients over 50-year-old referred to the Kermanshah Health Bus.

The findings of this study showed that the highest incidence of musculoskeletal disorder was in the knee. Then, the incidence of the low back had the most frequency among men and women above 50-year-old.

In a study by Hartvigsen et al. the highest musculoskeletal pain in the population above 45-year-old was observed in the low back, neck, shoulder, and knee [17] which are in line with our results.

The results of the present study showed that the lowest incidence of musculoskeletal disorders in the population above 50-year-old was related to the thigh/hip area. In the study of Hartvigsen et al., the thigh area was also one of the areas with the lowest pain reporting by the population [17]. The highest incidence of the musculoskeletal disorders in different parts of the body of males and females, both over the last week and the last year, was related to the low back region. The results of the study by Hartvigsen et al. and Kweku Nakua also indicated that the low back area in both genders had the highest levels of pain [17]. The study of Wijnhoven et al. showed that in the Dutch population between 25 and 64, the least difference in the prevalence of musculoskeletal problems in both genders was related to the low back pain and knee pain, which is similar to our study. It seems that low back pain and knee pain are the most common musculoskeletal disorders in the community [18].

Most people referred to the health bus were women, either due to more musculoskeletal problems among them, or because men due to their job had less free time during the health bus assessment (before noon) and could not refer to assess their health status.

In the mentioned studies, the higher occurrence of musculoskeletal problems in women has been confirmed. The study of Wijnhoven et al. showed that the prevalence of these problems among Dutch people in the age range of 25–64 was higher in women than men [18]. The results of the recent study showed that the prevalence of musculoskeletal disorders among housewives was related to the low back and knee. Since repetitive movements and staying in a static position for a long time are the main reasons for musculoskeletal pain [19], probably doing the housework by Iranian women who are usually housewives may cause more problems. To justify the increased prevalence of these problems in women, three kinds of reasons are presented: 1- women express their pain more than men 2- due to hormonal and physiological differences, women are more vulnerable to musculoskeletal problems than men 3- women are more exposed to risk factors of developing musculoskeletal problems than men [18].

The findings of this study showed that among the participants of the study, most workplace accidents led to musculoskeletal disorders in the low back area, and the lowest workplace accidents were related to the shoulder region. In the study of Ge et al. that was done on oil industry workers, low back disorders were most prevalent among the workers, followed by neck and shoulder problems [20].

Considering the educational level, the highest frequency was related to high school and the lowest was related to an academic degree, that these findings were similar to Picavet’s study [21]. Perhaps raising the education level will increase the awareness and prevention of musculoskeletal disorders.

## Limitations

Among the limitations of this study, it could be noted that the number of women participants was more than men, which may be due to the employment and working of men during the time of health bus working and lack of opportunity to refer the bus.

## Supplementary information

**Additional file 1:** Frequency distribution of the occurred accident in the workplace divided by body regions based on the age range of the participants.

**Additional file 2:** Frequency distribution of the prevalence of musculoskeletal disorders in different parts of the body in the last week and last year based on the in the participants’ BMI groups.

**Additional file 3:** The prevalence of musculoskeletal disorders in different parts of the body in the last year based on level of education.

## Data Availability

The datasets used and/or analyzed during the current study are available from the corresponding author on reasonable request.

## Abbreviations

BMI: Body mass index
MSDs: Musculoskeletal disorders
