# Supplementary material for: The prevalence of musculoskeletal pain among above 50-year-old population referred to the Kermanshah-Iran health bus in 2016
Source: BMC Res Notes. 2020 Feb 12;13:72. doi: 10.1186/s13104-020-4940-6 (PMC7017604; doi:10.1186/s13104-020-4940-6)

**Figure S1.** Frequency Distribution of the Occurred Accident in the Workplace Divided by Body Regions based on the Age Range of the Participants


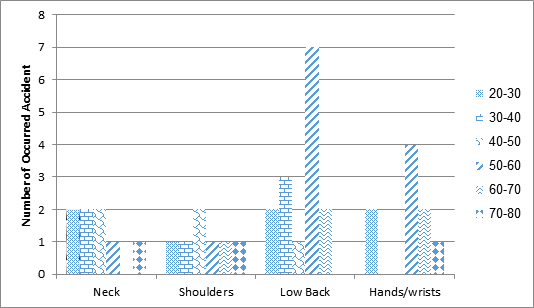

Supplement: Supplementary file 1 — Additional file 1: Frequency distribution of the occurred accident in the workplace divided by body regions based on the age range of the participants. [file 13104_2020_4940_MOESM1_ESM.docx]
