# Supplementary material for: The prevalence of musculoskeletal pain among above 50-year-old population referred to the Kermanshah-Iran health bus in 2016
Source: BMC Res Notes. 2020 Feb 12;13:72. doi: 10.1186/s13104-020-4940-6 (PMC7017604; doi:10.1186/s13104-020-4940-6)
Supplement: Supplementary file 2 — Additional file 2: Frequency distribution of the prevalence of musculoskeletal disorders in different parts of the body in the last week and last year based on the in the participants’ BMI groups. [file 13104_2020_4940_MOESM2_ESM.docx]

**Table S1 -** Frequency Distribution of the Prevalence of Musculoskeletal Disorders in Different Parts of the body in the last week and Last Year based on the in the Participants’ BMI Groups

| Body Parts | Having pain in recent 12 month (number (%)) | | | | | | | | Having pain in recent week (number (%)) | | | | | | | |
| --- | --- | --- | --- | --- | --- | --- | --- | --- | --- | --- | --- | --- | --- | --- | --- | --- |
|  | <18.5 | | 18.5-25 | | 25-30 | | BMI>30 | | <18.5 | | 18.5-25 | | 25-30 | | BMI>30 | |
|  | Yes | No | Yes | No | Yes | No | Yes | No | Yes | No | Yes | No | Yes | No | Yes | No |
| Neck | 4  (0.7%) | 5  (0.9%) | 47  (8.1%) | 79  (13.6%) | 86  (14.8%) | 156  (26.9%) | 86  (14.8%) | 117  (20.2%) | 1  (0.2%) | 8  (1.4%) | 40  (6.9%) | 86  (14.9%) | 72 (12.5%) | 170  (29.3%) | 74  (12.8%) | 129  (22.2%) |
| Shoulder | 4  (0.7%) | 5  (0.9%) | 54  (9.3%) | 72  (12.4%) | 87  (15%) | 155  (26.5%) | 95  (16.4%) | 108  (18.6%) | 2  (0.3%) | 7  (1.2%) | 41  (7.1%) | 85  (14.7%) | 74  (12.8%) | 168  (29%) | 75  (12.9%) | 128  (22.1%) |
| Elbow | 2  (0.3%) | 7  (1.2%) | 24  (4.1%) | 102  (17.6%) | 45  (7.8%) | 197  (34%) | 52  (9%) | 151  (26%) | 1  (0.2%) | 8  (1.4%) | 23  (4%) | 103  (17.8%) | 34  (5.9%) | 208  (35.9%) | 43  (7.4%) | 160  (27.6%) |
| Hand/Wrist | 2  (0.3%) | 7  (1.2%) | 39  (6.6%) | 87  (15%) | 74  (12.6%) | 168  (29%) | 72  (12.4%) | 131  (23%) | 2  (0.3%) | 7  (1.2%) | 35  (6%) | 91  (15.7%) | 62  (10.7%) | 180  (31%) | 61  (10.5%) | 142  (24.5%) |
| Upper Back | 2  (0.3%) | 7  (1.2%) | 37  (6.4%) | 89  (15.3%) | 85  (14.6%) | 157  (27.2%) | 71  (12.2%) | 132  (22.8%) | 2  (0.3%) | 7  (1.2%) | 30  (5.2%) | 96  (16.6%) | 67  (11.5%) | 175  (30.2%) | 55  (9.5%) | 148  (25.5%) |
| Lower Back | 6  (1%) | 3  (0.5%) | 61  (10.5%) | 65  (11.2%) | 133  (22.9%) | 109  (18.8%) | 115  (19.8%) | 88  (15.2%) | 6  (1%) | 3  (0.5%) | 55  (9.5%) | 71  (12.2%) | 116  (20%) | 126  (21.7%) | 100  (17.2%) | 103  (17.8%) |
| Hip Thigh/ | 2  (0.3%) | 7  (1.2%) | 18  (3.1%) | 108  (18.6%) | 38  (6.5%) | 204  (35.2%) | 34  (5.9%) | 169  (29.1%) | 2  (0.3%) | 7  (1.2%) | 14  (2.4%) | 112  (19.3%) | 29  (5%) | 213  (36.7%) | 28  (4.8%) | 175  (30.2%) |
| Knee | 5  (0.9%) | 4  (0.7%) | 62  (10.7%) | 64  (11%) | 135  (23.3%) | 107  (18.3%) | 129  (22.2%) | 74  (12.8%) | 4  (0.7%) | 5  (0.9%) | 47  (8.1%) | 79  (13.6%) | 94  (16.2%) | 148  (25.5%) | 103  (17.8%) | 100  (17.2%) |
| Ankle/Foot | 3  (0.5) | 6  (1%) | 21  (3.6%) | 105  (18.1%) | 60  (10.3%) | 182  (31.4%) | 63  (10.9%) | 140  (24.1%) | 3  (0.5%) | 6  (1%) | 18  (3.1%) | 108  (18.6%) | 44  (7.6%) | 198  (34.1%) | 56  (9.7%) | 147  (25.3%) |
