# Supplementary material for: The prevalence of musculoskeletal pain among above 50-year-old population referred to the Kermanshah-Iran health bus in 2016
Source: BMC Res Notes. 2020 Feb 12;13:72. doi: 10.1186/s13104-020-4940-6 (PMC7017604; doi:10.1186/s13104-020-4940-6)
Supplement: Supplementary file 3 — Additional file 3: The prevalence of musculoskeletal disorders in different parts of the body in the last year based on level of education. [file 13104_2020_4940_MOESM3_ESM.docx]

Table S2. The prevalence of musculoskeletal disorders in different parts of the body in the last year based on level of education

|  | Illiterate | Non-diploma | Diploma | Upper-diploma | p-value |
| --- | --- | --- | --- | --- | --- |
| Neck | 86 (46.7) | 39(35.1) | 16(34) | 5(16.1) | 0.006 |
| Shoulder | 90 (48.9) | 51 (45.9) | 15 (31.9) | 7 (22.6) | 0.015 |
| Elbow | 51 (27.7) | 32 (28.8) | 6 (12.8) | 4 (12.9) | 0.51 |
| Wrist | 78 (42.4) | 35 (31.5) | 11 (23.4) | 7 (22.6) | 0.20 |
| Back | 54 (29.3) | 28 (25.2) | 4 (8.5) | 6 (19.4) | 0.26 |
| Hip | 91 (49.5) | 58 (52.3) | 21 (44.7) | 12 (38.7) | 0.543 |
| Thigh | 38 (20.7) | 13 (11.7) | 8 (17) | 4 (12.9) | 0.228 |
| Knee | 141 (76.6) | 63 (56.8) | 21 (44.7) | 11 (35.5) | 0.001 |
| Foot | 60 (32.6) | 27 (24.3) | 14 (29.8) | 6 (19.4) | 0.288 |

Frequency (%) was reported/ p-value based on chi-square test.
